# PEDV Infection Generates Conformation-Specific Antibodies That Can Be Effectively Detected by a Cell-Based ELISA

**DOI:** 10.3390/v13020303

**Published:** 2021-02-15

**Authors:** Wei-Ting Hsu, Chia-Yu Chang, Chih-Hsuan Tsai, Sung-Chan Wei, Huei-Ru Lo, Robert John S. Lamis, Hui-Wen Chang, Yu-Chan Chao

**Affiliations:** 1 Institute of Molecular Biology, Academia Sinica, Taipei 115, Taiwan; waitinglovekeroro@gmail.com (W.-T.H.); lt22448@gmail.com (C.-H.T.); nerv.lilith@gmail.com (S.-C.W.); hrl@gate.sinica.edu.tw (H.-R.L.); robertjohnlamis@yahoo.com (R.J.S.L.); 2 Graduate Institute of Life Sciences, National Defense Medical Center, Taipei 114, Taiwan; 3 School of Veterinary Medicine, National Taiwan University, Taipei 106, Taiwan; flywinds11@gmail.com (C.-Y.C.); huiwenchang@ntu.edu.tw (H.-W.C.); 4 Molecular and Cell Biology, Taiwan International Graduate Program, Academia Sinica and Graduate Institute of Life Science, National Defense Medical Center, Taipei 115, Taiwan; 5 Graduate Institute of Molecular and Comparative Pathobiology, School of Veterinary Medicine, National Taiwan University, Taipei 106, Taiwan; 6 Department of Entomology, National Chung Hsing University, Taichung 402, Taiwan; 7 Department of Plant Pathology and Microbiology, College of Bioresources and Agriculture, National Taiwan University, Taipei 106, Taiwan

**Keywords:** baculovirus, cell-based ELISA, coronavirus, PEDV, spike

## Abstract

Porcine epidemic diarrhea virus (PEDV) is a coronavirus that causes serious and highly contagious enteric disease in swine worldwide. In this study, we constructed a recombinant baculovirus (S-Bac) expressing full-length spike protein of the virulent epidemic genotype 2b (G2b) PEDV strain for serological studies of infected pigs. We found that most spike-specific antibodies produced upon PEDV infection in pigs are conformation-specific and they could be detected on S-Bac-infected insect cells by immunofluorescent assay, but they were insensitive to Western blot analysis, the typical method for antiserum analysis. These results indicated that spike conformation is crucial for serum recognition. Since it is difficult to purify trimeric spike membrane protein for conventional enzyme-linked immunosorbent assay (ELISA), we used S-Bac to generate a novel cell-based ELISA for convenient PEDV detection. We analyzed 100 pig serum samples, and our cell-based ELISA exhibited a sensitivity of 100%, a specificity of 97%, and almost perfect agreement [Cohen’s kappa coefficient value (κ) = 0.98] with immunocytochemical staining results. Our cell-based ELISA rapidly presented antigen for proper detection of conformation-specific antibodies, making PEDV detection more convenient, and it will be useful for detecting many viral diseases in the future.

## 1. Introduction

Porcine epidemic diarrhea virus (PEDV) is a member of the genus *Alphacoronavirus* of the family *Coronaviridae*, which has been associated with highly infectious enteropathy in pigs of all ages, causing acute diarrhea, dehydration, and vomiting. The infection causes mild disease in adult pigs, but causes nearly 100% mortality in neonatal piglets, leading to significant economic losses for global pork industries in recent years [1,2,3]. PEDV was first reported in England around the early 1970s, but later spread to the rest of Europe and Asia [3,4]. In 2013–2014, a new highly virulent strain was introduced into North America and subsequently spread to Canada and Mexico [1,2,5,6,7]. The strain has also been reported from South Korea and Taiwan [8,9]. In 2014, we isolated a highly virulent PEDV Pingtung 52 (PEDV-PT) strain from a farm in Pingtung, Taiwan, and identified it as genogroup 2b (G2b) [10].

PEDV contains a positive-sense, single-stranded RNA genome of approximately 28 kb [11,12]. The PEDV genome encodes four structural proteins––spike (S), nucleocapsid (N), envelope (E), and membrane (M)––as well as three other ORFs, namely, ORF1a, ORF1b, and ORF3, encoding non-structural proteins [2,3,11,12,13,14]. During outbreaks, reverse transcription-polymerase chain reaction (RT-PCR) systems are generally employed for PEDV detection, informing quarantine and slaughter policies [2]. However, the virus-shedding interval is short. In order to effectively monitor PEDV epidemiology to prevent piglet infection, serological analysis is necessary.

PEDV S protein is the most appropriate viral antigen for developing effective vaccines and diagnostic assays [5,15,16,17]. S protein is a glycoprotein of ~1383 residues and 180–220 kDa [18,19]. It forms club-shaped trimers of ~20 nm extending from the virion surface, giving this coronavirus the typical crown-like appearance observed via electron micrography [4]. S protein mediates the essential functions of receptor binding and subsequent fusion of the viral and cellular membranes during cell entry [12,18,20,21,22]. Like other coronavirus S protein monomers, PEDV S can also be divided into two subunits: the N-terminal S1 subunit for receptor binding and the C-terminal membrane-anchored S2 subunit for membrane fusion [18]. The detailed monomer structure contains an N-terminal signal peptide, a vast extracellular region, a single transmembrane (TM) domain, and a short cytoplasmic tail domain (CTD) [18,19].

**Figure 1:**
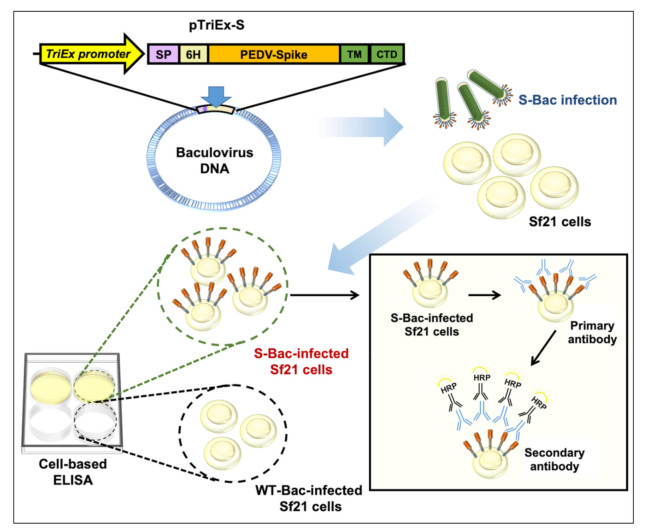
Schematic showing how S-Bac-infected cells are used for cell-based ELISA. S-Bac was used to infect Sf21 cells to display S on the cell membrane. These S-displayed cells, together with WT-Bac-infected cells (as control cells), were used to establish a cell-based ELISA system. The presence of anti-S primary antibodies is detected by horseradish peroxidase (HRP)-conjugated secondary antibody and can be quantified based on subsequent color development.

S is a suitable antigen target for developing serological assays [23]. In previous studies, several PEDV anti-S monoclonal antibodies (mAb) having virus infection-neutralizing activity were found to target the conformational epitopes rather than linearized epitopes on S protein [24,25,26]. Interaction of these conformation-specific antibodies with S could not be readily detected by Western blot or dot blotting, but was strongly detected via immunofluorescence analysis (IFA) using PEDV-infected cells displaying authentic S proteins [24,25,26]. These observations suggest that an S-based detection system using segmented or denatured antigens may fail to detect neutralizing antibodies. However, the experimental protocols for IFA are tedious and it can be difficult to quantify the results. An ELISA-based method would offer a more convenient approach to examining pig sera. Previously, we performed ELISA studies using purified S proteins and found that this approach detected the serum satisfactorily [23]. However, S is a highly glycosylated protein, so it is challenging to express it in prokaryotic systems. In addition, S is a trimeric transmembrane protein, so it is difficult to express and purify it even in mammalian systems. The trimeric conformation of S may also be damaged during the purification process, thereby inhibiting antibody recognition. To solve these problems, we developed a cell-based ELISA whereby S protein is displayed on insect cells as an antigen (Figure 1).

In a previous study, we displayed PEDV S protein on the envelope of baculovirus as an efficient vaccine to prevent PEDV infection in swine [27]. Baculovirus expression systems have long been used as a tool for foreign protein expression while maintaining proper eukaryotic glycosylation and other post-translational modifications [28,29]. Such systems possess the appropriate machinery for mammalian protein folding and, consequently, are suitable for producing soluble proteins of mammalian origin [30]. Baculoviruses are a family of giant, rod-shaped, enveloped viruses possessing large, circular, double-stranded DNA genomes (80–180 kbp), with typical sizes of 40–50 nm in diameter and 200–400 nm in length [31]. Baculoviruses infect a diverse array of invertebrates, especially insects, but not mammals [31,32,33]. The most widely studied and commonly used baculovirus for expression studies is Autographa californica multiple nucleopolyhedroviruses (AcMNPV), which infects lepidopteran species (e.g., *Spodoptera frugiperda*). Cell lines such as Sf21 are used to propagate AcMNPV [28]. The major glycoprotein GP64 of AcMNPV has been reported as a suitable fusion partner for foreign protein surface displays [29].

Here, we generated a recombinant baculovirus (S-Bac) expressing the PEDV-S ectodomain fused with the TM and CTD of GP64. By using S-Bac-infected insect cells, we displayed a high level of recombinant S protein on insect cell membranes as an antigen for PEDV serological analyses. Surprisingly, we found that sera from PEDV-infected pigs did not interact well with the S antigen in Western blots, likely due to the fact that PEDV infection mainly generates conformation-specific antibodies. Nevertheless, our cell-based ELISA using S-Bac-infected insect cells could determine PEDV infection in serum samples from infected specific pathogen-free (SPF) piglets and swine from pig farms. Since our cell-based ELISA does not require protein purification and immobilization steps, it maintained the polymeric S protein conformation for conformation-specific antibody detection and represents a convenient and efficient diagnostic tool for PEDV infection.

## 2. Materials and Methods

### 2.1. Cell Lines and Cell Culture

Vero cells and *Spodoptera frugiperda* IPLB-Sf21 (Sf21) were cultured as previously described [34,35]. In brief, Vero cells were cultured with MEM-alpha medium (Thermo Fisher Scientific, Waltham, MA, USA) containing 10% fetal bovine serum (FBS) and maintained in a humidified incubator with 5% CO_2_ at 37 °C, whereas Sf21 cells were cultured in TC100 insect medium (Thermo Fisher Scientific, Waltham, MA, USA) with 10% FBS at 26 °C.

### 2.2. Plasmid Construction

**Figure 2:**
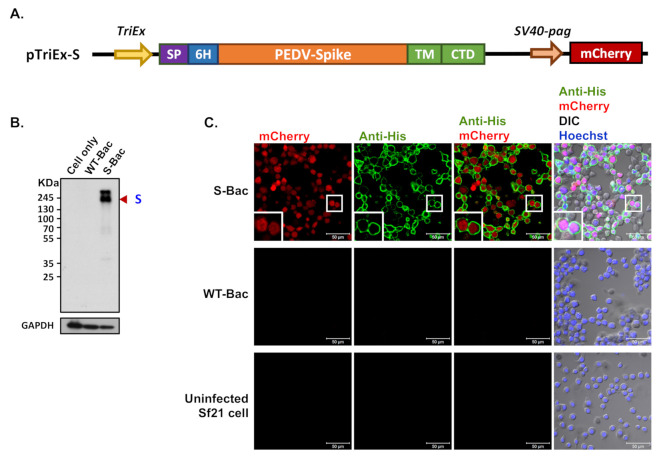
Determination of recombinant PEDV S protein expression by Western blot and IFA. (**A**) The construction map of the pTriEx-S plasmid. The full-length *S* gene of PEDV was cloned into pTriEx-4 plasmid to form pTriEx-S. The TM and CTD regions of the S protein were replaced with those of GP64 protein from baculovirus for proper membrane protein display. Expression of the S construct was driven by the *TriEx* promoter (*TriEx*), which was followed by the honeybee melittin signal peptide (SP) and 6X His tag (6H). An mCherry reporter driven by the *SV40-pag* promoter (*SV40-pag*) was also incorporated into pTriEx-S. (**B**) Western blot analysis of the recombinant S protein. S protein was expressed upon infection of S-Bac in insect cells and subjected to Western blot analysis. Cell only: uninfected Sf21 cells; WT-Bac: Sf21 cells infected with WT-Bac; S-Bac: Sf21 cells infected with S-Bac; GAPDH: cellular protein as a loading control. Primary antibody: anti-His antibody with a dilution of 1:5000. Secondary antibody: goat anti-mouse IgG conjugated to HRP with a dilution of 1:5000. (**C**) IFA of S protein on the infected insect cells. S protein presence was confirmed by anti-His antibody immunofluorescence (green fluorescence) on Sf21 cells infected with S-Bac or WT-Bac, and uninfected Sf21 cells. All samples were counterstained with Hoechst 33342 dye (blue). DIC: differential interference contrast microscopy. Insets: magnifications of the single cell marked by white boxes. Primary antibody: anti-His antibody with a dilution of 1:5000. Secondary antibody: Alexa Fluor 488 goat anti-mouse IgG with a dilution of 1:200. Bar: 50 µm.

The nucleotide sequence of the *S* (Genbank accession No. KP276252) and *N* (Genbank accession No. ART85703.1) genes derived from the Taiwan G2b PEDV-PT strain was synthesized by ProTech (ProTech, Taipei, Taiwan), as previously described [27,36]. For protein expression, the *S* and *N* genes were codon-optimized for insect cell expression. The ectodomain of S and the full-length N were cloned into TriEx-4 plasmid (Millipore, Burlington, MA, USA) with a honeybee melittin signal peptide, a 6xHis-tag, and GP64 TM and CTD (Figure 2A) to generate plasmids pTriEx-S and pTriEx-N, respectively. The binary *SV40*-*pag* promoter [37] was used to drive mCherry expression for reporter fluorescence in Sf21 cells. The plasmids were constructed according to the instruction manual of the In-Fusion® HD Cloning Kit (Clontech Laboratories Inc., Fremont, CA, USA).

### 2.3. Recombinant Baculovirus Preparation

The pTriEx-S and pTriEx-N plasmids were co-transfected with FlashBAC™ (Mirus, Madison, WI, USA) into Sf21 cells using Cellfectin (Thermo Fisher Scientific, Waltham, MA, USA) to generate S-Bac and N-Bac recombinant baculoviruses. After incubation at 26 °C for five days, virus stocks were harvested from the culture supernatants. Single viruses were isolated by serial dilution, with viral infection and protein expression tracked by mCherry reporter expression and 6xHis-tag signal, respectively. Single viruses with better target protein expression were amplified and the titers were determined according to the 50% tissue culture infection dose (TCID_50_).

### 2.4. Virus Titer Determination by TCID_50_

Sf21 cells (4 × 10^4^) were seeded on a 96-well plate and incubated at room temperature (26 °C) for at least 1–2 h for complete attachment. Viral solutions were diluted (10^−1^~10^−10^) in TC100 medium supplemented with 10% FBS. Medium in each well of the 96-well plate was replaced with 100 µL of virus solution from each dilution. Each dilution was repeated 16 times. For efficient virus infection, plates were centrifuged at 2000 rpm for 30 min, and plates were placed in an incubator at 26 °C for five days before observation. Virus titer was determined by counting the number of wells exhibiting infection for each dilution of the virus.

### 2.5. Western Blot Analysis

Sf21 cells were infected with S-Bac or WT-Bac using an multiplicity of infection (M.O.I.) of 5 and incubated at 26 °C for three days. Uninfected or infected insect cells were lysed in sample buffer by boiling for 10 min and subjected to sodium dodecyl sulfate (SDS)-polyacrylamide electrophoresis (PAGE). The samples were resolved on a 10% SDS-PAGE gel in running buffer (200 mM glycine, 1% SDS, 2.5 mM Tris/HCl). After being resolved, samples were transferred to a polyvinylidene fluoride (PVDF) membrane with transfer buffer (10% methanol, 192 mM glycine, 25 mM Tris) at 300 mA at 4 °C for 90 min. The PVDF membrane containing protein samples was washed in phosphate-buffered saline (PBS) and blocked using 3% BSA in PBS for 1 h at room temperature. To detect S or N protein expression levels, the membrane was hybridized with the primary antibody (mouse anti-6X His-tag mAb, 1:5000 dilution, EnoGene, New York, NY, USA) followed by the secondary antibody goat anti-mouse IgG antibody (1:5000 dilution, Thermo Fisher Scientific, Waltham, MA, USA). To detect antibody contents in swine sera, the membrane was hybridized with the serum sample (1:2000 dilution) followed by goat anti-swine IgG (1:5000 dilution, Invitrogen, Carlsbad, CA, USA). To analyze interaction with P4B antibody, the membrane was hybridized with blocking buffer containing P4B (1:1000 dilution) followed by the secondary antibody goat anti-mouse IgG antibody (1:5000 dilution, Thermo Fisher Scientific, Waltham, MA, USA). Protein bands were visualized using Clarity™ Western ECL Blotting Substrates (Bio-Rad, Hercules, CA, USA) and Classic Blue Autoradiography film BX (Thermo Fisher Scientific, Waltham, MA, USA).

### 2.6. Detection of Cell Surface Protein by IFA

IFA was performed using a previously established protocol [34,38]. Briefly, Sf21 cells (2 × 10^5^) were seeded onto eight-well Millicell® EZ slides (Millipore, Burlington, MA, USA) and infected with S-Bac or WT-Bac using an M.O.I. of 1 and incubated at 26 °C for 48 h. The cells were treated with 4% paraformaldehyde for 10 min on ice for fixation. After three washes with DPBST (Dulbecco’s phosphate-buffered saline, DPBS, plus 0.1% Tween 20), the cells were blocked with 3% BSA for 1 h and then incubated with primary antibody overnight at 4 °C. Protein signals were detected using mouse anti-6X His-tag mAb (1:5000 dilution, EnoGene, New York, NY, USA), P4B antibody (1: 200 dilution), and pig serum samples (1: 200 dilution). After overnight incubation, cells were washed three times with DPBST and incubated with Alexa Fluor 488 goat anti-mouse IgG secondary antibody (Thermo Fisher Scientific, Waltham, MA, USA) or Alexa Fluor 488 goat anti-swine IgG (Thermo Fisher Scientific, Waltham, MA, USA) at a dilution of 1:200. Images (1024 × 1024 diameter pixels) were obtained using a Zeiss laser confocal microscope (LSM780) with a Fluor 63×/1.40 NA oil-immersion objective, and fluorescence intensity was determined in ZEN 2010 software (Zeiss).

### 2.7. Specimen Information

A total of 100 serum samples, including 37 negative controls, 37 positive controls, and 26 experimental samples, were assessed. The 37 negative controls without a history of PEDV exposure included 14 samples from post-weaning SPF piglets purchased from the Animal Technology Institute of Taiwan (ATIT) and 23 samples from piglets reared in a highly managed conventional farm. We orally challenged these negative control piglets with 5 × 10^5^ TCID_50_ G2b PEDV-PT strain. Serum samples collected two weeks after PEDV challenge represented the positive samples. In addition, sera collected from 26 sows fed intestinal homogenate from G2b PEDV-infected piglets were used as the experimental groups. These serum samples were named as follows: specific pathogen-free PEDV-negative (SPF-N) piglets, conventional farm-derived PEDV negative (C-N) piglets, PEDV-inoculated SPF (SPF-I) piglets, PEDV-inoculated conventional farm (C-I) piglets, and farm-based sows fed with PEDV-positive intestinal homogenate (Sow).

### 2.8. PEDV-Infected Vero Cells

Vero cells infected with G2b PEDV-PT strain (GenBank: KY929405) were used as ICC antigen, as previously described [10,39]. Briefly, Vero cells were seeded in 96-well plates and then inoculated with 1000 TCID_50_/mL of PEDV-PT diluted in a post-inoculation medium containing DMEM (Thermo Fisher Scientific, Waltham, MA, USA), 10 μg/mL trypsin (Thermo Fisher Scientific, Waltham, MA, USA), 0.3% tryptose phosphate broth (Millipore, Burlington, MA, USA), and 0.02% yeast extract (Millipore, Burlington, MA, USA). After 24 h of incubation, we assessed the cytopathic effect (CPE. i.e., syncytial cells) for all Vero cells, and those cells exhibiting CPE coverage of at least 10% were selected. After removal of the supernatant, the cells were treated with 80% acetone (Millipore, Burlington, MA, USA) and fixed for 20 min. After discarding the acetone, the cells were air-dried for 1 h and then stored at −20 °C.

### 2.9. Immunocytochemical (ICC) Staining

Anti-G2b PEDV-PT titers of the 100 serum samples described above in *Specimen information* were characterized by ICC staining [23]. All serum samples were two-fold serially diluted (1:160 to 1:20,480), added to plates fixed with PEDV-infected Vero cells, and incubated for 1 h at 26 °C. After supernatants were discarded, the cells were washed with PBS at least six times. HRP-conjugated anti-pig IgG antibodies were added to detect the binding serum IgG on the syncytial cells. After incubation for 1 h, a 3,3′-diaminobenzidine (DAB) coloration system was used to visualize signals. ICC titer was determined as the reciprocal of the highest dilution with coloring signal. ICC titers <160 were considered as staining backgrounds. All negative samples, including SPF-N and C-N piglets, had ICC titers <160, so they were confirmed to be negative serum samples and their titers are shown as 0.

### 2.10. Establishment of Cell-Based ELISA by S-Bac Infection of Sf21 Cells

Sf21 cells were infected with S-Bac or WT-Bac using an M.O.I. of 1 and incubated at 26 °C for 48 h. Insect cells, i.e., S-Bac-infected cells or WT-infected cells, were washed with PBS and seeded in the well of a 96-well microplate (1 × 10^4^/well) for 1 h at room temperature. The cells were fixed with 4% paraformaldehyde for 10 min, washed three times with PBST (PBS with 0.1% Tween 20), then blocked with 3% BSA for 1 h. Cells were incubated with diluted antisera for 2 h at 26 °C. After incubation, cells were washed three times with PBST and incubated with Peroxidase AffiniPure goat anti-swine IgG (1:5000 dilution, Jackson ImmunoResearch) as secondary antibody for signal detection following reaction with 3,3′,5,5′-Tetramethylbenzidine (TMB) substrate for 1 h. The plates were read at 450 nm using an EnSpire Series Multilabel Plate Reader (PerkinElmer). Mean optical density (OD) values were used to quantify ELISA data.

### 2.11. Determination of Cutoff Value, Specificity, and Sensitivity of S-Bac Cell-Based ELISA

We deployed a general formula to determine the cutoff values for ELISA, which is based on the mean value of the negative samples (SPF-N and C-N) plus two or three standard deviations (mean +2 SD or mean +3 SD). Sensitivity and specificity were evaluated against ICC staining results, in which sensitivity calculates the proportion of correctly identified ICC positives and specificity calculates the proportion of correctly identified ICC negatives. The receiver operating characteristic (ROC) analysis was used to analyze the discriminant capacity of a specified cutoff value. The ROC curve was obtained by plotting the Sensitivity (*Y*-axis) against 1-Specificity (*X*-axis).

### 2.12. Statistical Analysis

Cohen’s kappa coefficient values (𝜅) were calculated for ICC staining and S-Bac-infected cell-based ELISA to assess the inter-rater agreement. Strength of agreement was considered poor if 𝜅 ≤0, slight for 0.01–0.20, fair for 0.21–0.40, moderate for 0.41–0.60, substantial for 0.61–0.80, and almost perfect for 0.81–1. Repeatability of our S-Bac-infected cell-based ELISA was assessed by running all serum samples independently three times, and mean OD value for each sample is presented.

## 3. Results 

### 3.1. Construction of S-Bac and Detection of S Protein Expression in Infected Insect Cells

Recombinant S-Bac virus was constructed by fusing the S protein ectodomain of PEDV-PT with the TM and CTD regions of GP64 (Figure 2A), thereby properly anchoring the entire construct on the plasma membrane of insect cells for protein display. A honeybee melittin signal peptide [29] and a 6X His-tag were added to the N-terminus of the construct (Figure 2A) to secrete the protein construct extracellularly and label the expressed proteins, respectively. In order to evaluate expression of the recombinant S protein, Sf21 insect cells were infected with S-Bac or wild-type AcMNPV virus (WT-Bac), and the lysed cells were analyzed by Western blot using antibodies against the incorporated 6X His-tag. Positive S protein signals were observed at ~180 kDa in the S-Bac-infected cell lysate (Figure 2B). S protein was not detected in the lysates of non-infected Sf21 cells or WT-Bac-infected Sf21 cells, indicating specific S expression in S-Bac-infected cells (Figure 2B).

### 3.2. Spike Protein Displays on Insect Cell Membranes and Is Properly Detected by IFA

We performed IFA to determine the localization of S in S-Bac-infected Sf21 cells. Sf21 cells were infected by S-Bac with a M.O.I. of 1 and harvested at 48 h post-infection for immunofluorescent staining. Red fluorescence signal reflected mCherry reporter expression driven by the *pag* promoter, identifying the infected cells. Green fluorescence signal (anti-His) revealed S protein expression on insect cells upon S-Bac infection. There was no detectable red or green fluorescence signal in non-infected Sf21 cells or WT-Bac-infected Sf21 cells. In contrast, an apparent green fluorescence signal was distributed on the plasma membrane of S-Bac-infected cells, surrounding red fluorescence signal inside the cells (Figure 2C). These results indicate that S-Bac infection can lead to surface display of S on infected insect cells.

### 3.3. ICC Staining of Antisera against G2b PEDV-PT-Infected Vero Cells

Previously, we performed ICC staining to assess pig antisera with or without G2b PEDV-PT-infection [23]. Here, ICC was also used to characterize infection in serum samples. We collected PEDV-negative serum samples from 37 piglets, comprising sera from 14 uninfected SPF piglets (SPF-N) and 23 piglets from conventional farms without a PEDV exposure history (C-N), representing PEDV-negative controls for ICC experiments. These 37 piglets were then infected with PEDV to induce antiserum development, and the subsequent sera, 14 infected SPF piglets (SPF-I) and 23 infected conventional piglets (C-I), were used as infection-positive controls. For the experimental groups, we collected serum samples from 26 sows (Sow) in conventional farms that had been fed with intestinal homogenate from G2b PEDV-infected piglets for convenient immunization [23].

Anti-G2b PEDV-PT titers of all the sera were characterized by ICC staining following two-fold serial dilution. ICC serum titers for positive control or experimental groups––14 SPF-I, 23 C-I, and 26 Sow––ranged from 640 to 20,480, representing significant positive infection. ICC serum titers of all negative control samples, including C-N piglets and SPF-N piglets, were less than 160 (the reciprocal of the lowest serum dilution), confirming negative infection.

### 3.4. Western Blot Analysis of Pig Sera Fails to Detect Anti-PEDV S Antisera

**Figure 3:**
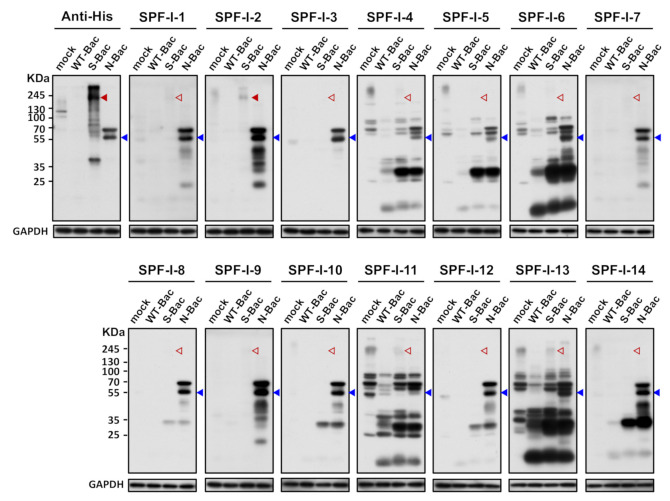
Western blot analysis reveals weak/absent anti-S antibody signals in PEDV-positive piglet sera. Uninfected Sf21 cells (mock) and Sf21 cells infected with WT-Bac, S-Bac, and N-Bac were subjected to Western blot analysis using piglet antisera. Primary antibody: anti-His antibody with a dilution of 1:5000 or PEDV-positive antiserum, SPF-I, with a dilution of 1:2000. Secondary antibody: goat anti-mouse IgG conjugated to HRP (1:5000 dilution) or goat anti-swine IgG conjugated to HRP (1:5000 dilution). Arrowheads: red, anti-S signal; blue, anti-N signal. Empty arrowheads represent an apparent lack of or weak anti-S signal.

Since anti-His antibody hybridization indicated proper S protein expression in S-Bac-infected insect cells (Figure 2), it represented a good platform for anti-S antibody analysis. We first selected the 14 SPF-I sera to study their antibody expression by Western blot. Unexpectedly, anti-S antibodies were barely detectable in these 14 PEDV-positive antisera when analyzed by Western blot (Figure 3, red arrowheads). In order to confirm that this outcome was not due to an improper Western blot process, we used the insect cells infected with the PEDV-N protein-expressing baculovirus (N-Bac) as a positive control for serum hybridization. We observed strong anti-N signals on the same blots using pig sera (Figure 3, blue arrowheads). These data indicated that PEDV infection may not efficiently induce antibodies against S protein or the majority of anti-S antibodies were conformation-specific antibodies that only recognized the non-denatured S protein conformation and, therefore, could not be detected in a typical denatured Western blot analysis.

### 3.5. IFA on S-Bac-Infected Cells Identifies Anti-PEDV S Antisera

**Figure 4:**
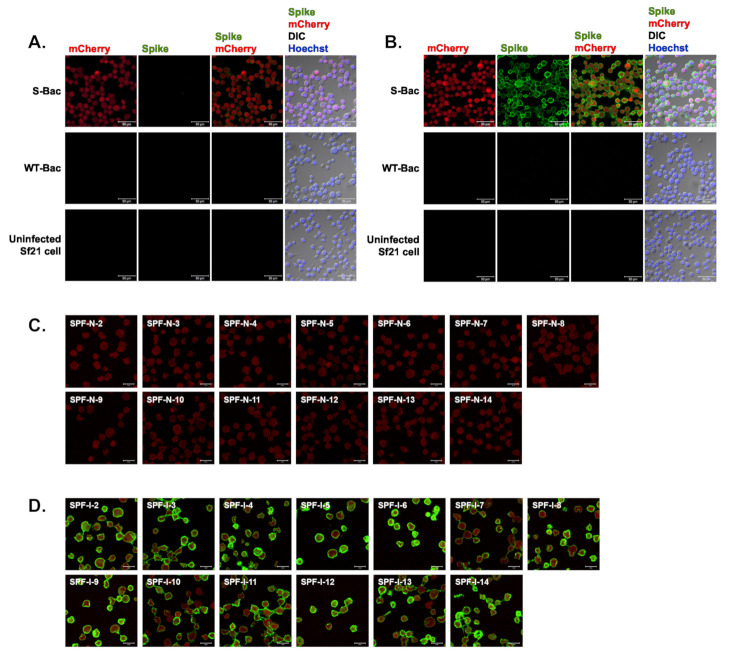
IFA of piglet antisera using Sf21 cells infected with S-Bac, WT-Bac, or uninfected Sf21 cells. Serum from SPF-N-1 (**A**) and SPF-I-1 (**B**) was used as primary antibody to interact with the cells, and the anti-S signal was detected by a secondary anti-swine green fluorescent antibody. For SPF piglets Nos. 2–14, only the merged image of anti-S (spike, green) and mCherry (red) signals are shown. DIC: differential interference contrast microscopy. (**C**) SPF-N, Nos. 2–14 (**D**) SPF-I, Nos. 2–14. Primary antibody: SPF-N or SPF-I serum with a dilution of 1:200. Secondary antibody: Alexa Fluor 488 goat anti-swine IgG with a dilution of 1:200. Bar: 50 µm.

To verify the existence of anti-S antibodies in pig sera, we conducted IFA with S-Bac-infected cells to analyze the SPF-N and SPF-I sera. Serum from SPF-N-1 (uninfected SPF piglet No. 1) did not interact with S protein on insect cell surfaces (Figure 4A), whereas Serum from SPF-I-1 (infected SPF piglet No. 1) did (Figure 4B). There was no signal detected in either WT-Bac-infected Sf21 cells or uninfected Sf21 cells (Figure 4A,B), indicating correct anti-S signal from S-Bac-infected cells. As exemplified by SPF-N-1 and SPF-I-1, sera from the remaining 13 SPF piglets before and after PEDV infection were assessed by IFA. The 13 SPF-N piglets did not show anti-S (green) on the surface of S-Bac-infected cells (Figure 4C), whereas the 13 SPF-I piglets did (Figure 4D). This analysis indicated that our piglets generated anti-S antibodies upon PEDV infection, which were not detectable by Western blot analysis but were fully detectable by IFA.

### 3.6. A Conformation-Specific Anti-S Antibody May Not Recognize S in a Denatured Western Blot Analysis

**Figure 5:**
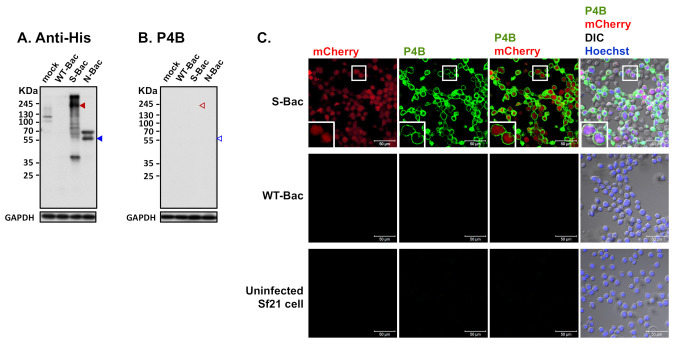
Detection of PEDV S protein by a neutralizing mAb, P4B. (**A**) Western blot analysis using anti-His antibody in uninfected Sf21 cells (mock) and in insect cells infected by WT-Bac, S-Bac, and N-Bac. Anti-His antibody detected the expression of S (red arrowhead) and N (blue arrowhead) in insect cells infected with S-Bac and N-Bac, respectively. Primary antibody: anti-His antibody (1:5000). Secondary antibody: goat anti-mouse IgG conjugated to HRP (1:5000). (**B**) Western blot analysis using P4B antibody in the same sample set of Figure 5A. Primary antibody: P4B antibody (1:1000). Secondary antibody: goat anti-mouse IgG conjugated to HRP (1:5000). (**C**) IFA to determine hybridization of P4B antibody with Sf21 cells infected with S-Bac, WT-Bac, and uninfected Sf21 cells. Signals of mCherry (derived from S-Bac infection), P4B antibody (green), and Hoechst counterstaining (blue) are shown. DIC: differential interference contrast microscopy. Insets: magnifications of the single cell marked by white boxes. Primary antibody: P4B antibody (1:1000). Secondary antibody: Alexa Fluor 488 goat anti-mouse IgG (1:200). Bar: 50 µm.

An anti-His-tag antibody could identify baculovirus clones expressing proper full-length S and N proteins in Western blot analysis (Figure 3 and Figure 5A). However, interestingly, the S-Bac-infected cells were not hybridized by a neutralizing mAb P4B against S1 [24] (Figure 5B). Previous study identified P4B as a conformation-specific antibody that only interacts with the natural conformation of S [24]. We examined whether P4B interacts with the S by IFA, behaving like SPF-I sera. We observed clear fluorescence signal of P4B (green) primarily located on the membrane of S-Bac-infected cells, whereas no detectable signal was observed in WT-Bac-infected or uninfected Sf21 cells (Figure 5C). This result illustrates that a neutralizing or conformation-specific anti-S antibody may only recognize the non-denatured conformation of S on authentic viral or cellular membranes, but not the protein in a denatured gel.

### 3.7. Establishment of a Novel Cell-Based ELISA Using S-Bac-Infected Insect Cells

**Figure 6:**
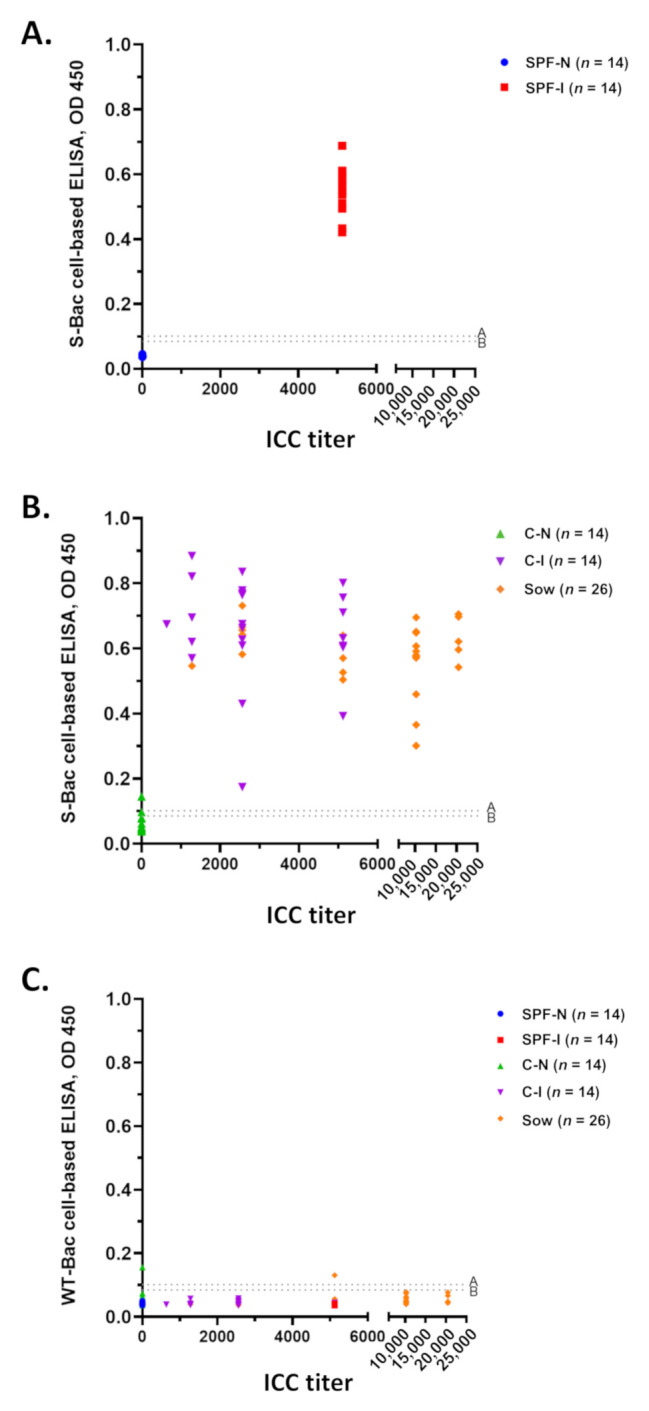
Serum detection using S-Bac-infected cell-based ELISA and its comparison to ICC titer. (**A**) Distribution of ICC titer and S-Bac-infected cell-based ELISA for SPF piglet sera, SPF-N and SPF-I. (**B**) Distribution of ICC titer and S-Bac-infected cell-based ELISA for piglet (C-N and C-I) and sow sera collected from conventional farms. (**C**) Comparison of ICC titer and WT-Bac-infected cell-based ELISA for all pig sera. ICC titers of each serum sample are presented according to serial dilution. Serum samples with ICC titer less than 160 are denoted as 0. Mean OD values represent the results of S-Bac-infected cell-based ELISA. The cutoff values were determined using the mean value for negative samples plus two or three standard deviations (SD). The dotted line A represents the cutoff value 0.1 (mean +3 SD) and dotted line B represents the cutoff value 0.09 (mean +2 SD). SPF-N: specific pathogen-free PEDV-negative piglets; SPF-I: PEDV-inoculated SPF piglets; C-N: conventional farm-based PEDV-negative piglets; C-I: PEDV-inoculated conventional piglets; Sow: farm-based sows fed with PEDV-positive intestinal homogenate.

Western blot analysis and IFA of SPF piglet sera (Figure 3 and Figure 4) and P4B antibody (Figure 5) indicated that PEDV infection in piglets mostly generated conformation-specific antibodies. These antibodies cannot be properly detected by Western blot but can be strongly detected by IFA using insect cells displaying authentic S proteins. For a convenient examination of pig sera, we seeded the S-Bac-infected cells into the wells of a 96-well microplate to act as the antigen for ELISA. We used this cell-based ELISA to examine each serum sample and compared the mean optical density (OD) of ELISA to ICC titers (Figure 6). Cutoff values were determined using the mean value of all the negative samples (14 SPF-N and 23 C-N) plus two or three standard deviations (SD), which were 0.09 (mean +2 SD) or 0.1 (mean +3 SD) (Figure 6, dotted lines A and B). For SPF piglets, all 14 serum samples from the SPF-I group (Figure 6A, red) were significantly positive compared to the 14 serum samples from the SPF-N group (Figure 6A, blue). For the piglets from conventional farms, all 23 serum samples from the C-I group were positive (Figure 6B, purple) and all but one of the C-N group (22/23) were negative at the cutoff of 0.1 (or 21/23 when the cutoff was 0.09) (Figure 6B, green). Furthermore, all the serum samples from the 26 sows fed with PEDV-positive intestinal homogenate were positive (Figure 6B, orange).

Since the S protein was not purified in our cell-based ELISA system, some background signals may be derived from the baculovirus-infected cells. To account for this potential bias, we assessed all 100 serum samples in parallel against cells infected with WT-Bac. We found that most of the serum samples exhibited low background signals, with only two serum samples (one from C-N and one from a sow) exhibiting signals slightly above the cutoff value of 0.1 (mean +3 SD) (Figure 6C). 

**Figure 7:**
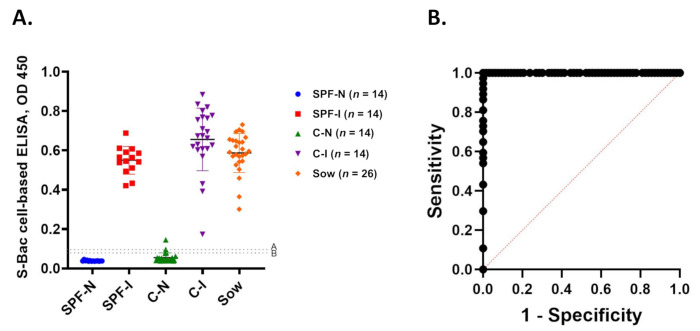
Summary of cell-based ELISA results and ROC analysis. (**A**) Summary of S-Bac-infected cell-based ELISA results for all 100 serum samples in each group in the study. (**B**) ROC curve of S-Bac-infected cell-based ELISA. Sensitivity (*Y*-axis) and 1-Specificity (*X*-axis) were calculated using the cutoff value 0.1 (mean +3 SD). SPF-N: specific pathogen-free PEDV-negative piglets; SPF-I: PEDV-inoculated SPF piglets; C-N: conventional farm-based PEDV-negative piglets; C-I: PEDV-inoculated conventional piglets; Sow: farm-based sows fed with PEDV-positive intestinal homogenate.

Positive and negative signals from S-Bac-infected cell-based ELISA are summarized in Figure 7A and Table 1. At a cutoff value of 0.09 (mean +2 SD), all SPF-N sera were negative, whereas all serum samples of the SPF-I, C-I, and sow groups were positive. Two of 23 C-N serum samples were positive, and the rest were negative. Accordingly, the sensitivity and specificity of our cell-based ELISA was 100% and 95%, respectively, with an almost perfect 𝜅 (agreement) of 0.96. When a cutoff value of 0.1 (mean +3 SD) was applied, the false-positive C-N serum samples were reduced to one, and the system could achieve 100% sensitivity and 97% specificity with an almost perfect 𝜅 of 0.98. The ROC curve derived by using the cutoff value 0.1 (mean +3 SD) revealed that this cutoff value had an excellent discriminant capacity (Figure 7B).

## 4. Discussion

In this study, we developed a novel, cell-based ELISA system based on S-Bac infection and demonstrated that it could distinguish sera from infected and healthy swine from different sources. This system improves on conventional indirect ELISA because it circumvents the need for antigen protein purification, which is especially tricky when these antigens are large, glycosylated, multimeric, or membrane-bound proteins. S antigen displayed on the insect cells correctly recognized the anti-S antibodies of PEDV-PT, and the majority of these anti-S antibodies were conformation-specific, which could not be detected by the denatured antigen. We believe this cell-based ELISA system can be a powerful tool to support the detection of other viral diseases.

Since as early as 2010, severe PEDV outbreaks in the pig population in China have caused enormous economic losses [6,39]. Although vaccines against classical PEDV vaccine strain CV777 are widely used in China, mortality from these outbreaks have been high, reaching 50–100% of neonatal piglets, indicating the emergence of new, highly virulent PEDV variants, such as the G2b PEDV strain currently circulating in North America and Asia [40,41,42]. Establishing diagnostic methods capable of detecting new PEDV infection is crucial for effective disease control and prevention. Many diagnostic assays have been developed for PEDV, including virus isolation, electron microscopy, immunohistochemistry tests, in situ hybridization, RT-PCR, IFA, and ELISA [2,7]. Among these technologies, ELISA is the most popular choice for serological testing to examine virus exposure histories and evaluate the efficacy of vaccinations [43]. ELISA-based systems have been developed that target PEDV proteins, including S, N, and M [44,45,46,47,48]. However, M- and N-based ELISA have been reported to cross-react with antibodies against other swine coronaviruses, and the current commercially available N-based test kit exhibits a low sensitivity of only 37% [23]. Therefore, we selected S as the target antigen for ELISA to detect G2b PEDV infection.

The S of PEDV is a large glycosylated trimeric transmembrane protein. Previously, we tried expressing truncated S to enhance protein expression in a HEK-293T system [23]. In this study, we adopted a different strategy by displaying the S protein on the insect cell membrane to circumvent the problematic issue of protein expression and purification. We generated the S-Bac recombinant baculovirus expressing full-length S of PEDV. Unlike the full-length construct used in our previous study for baculovirus envelope display [27], S-Bac in this study comprised the S ectodomain fused with GP64 TM and CTD to better anchor the protein on insect cell plasma membrane (Figure 2A). Western blotting revealed strong expression of the S construct in S-Bac-infected cells (Figure 2B), and IFA revealed that S protein was mainly displayed on cell surfaces (Figure 2C). The IFA using SPF-I sera (Figure 4C) and P4B antibody (Figure 5C) showed that anti-S antibodies can interact with the surface-displayed S, indicating our cell-based ELISA is a suitable system for pig serum detection.

Using this cell-based ELISA, we assessed a total of 100 serum samples, including 63 with and 37 without a PEDV infection history. Cutoff values according to the mean of ICC-negative sera plus 2 SD or 3 SD showed good discrimination. Signal from all ICC-positive sera was significantly higher than cutoff values, with only two or one C-N samples slightly exceeding the mean +2 SD or +3 SD thresholds, respectively (Figure 6C and Figure 7A). Moreover, the signal based on WT-Bac-infected cells was low (Figure 6C), consistent with the results of IFA using WT-Bac-infected cells to detect SPF-N-1 (Figure 4A), proving that neither the insect cells nor baculovirus infection contributed to background signal in our cell-based ELISA system. By using the cutoffs of mean +2 SD or +3 SD, our system exhibited 100% sensitivity, 95/97% specificity, and almost perfect agreement (𝜅 = 0.96 and 0.98) with ICC staining results (Table 1). These values are higher than those of our previously established full-length S-based ELISA (97.8% sensitivity, 94% specificity, and 𝜅 = 0.90), and similar to the results using S^1-501^ protein fragment, which is a truncated S optimized for ELISA (98.9% sensitivity, 99.1% specificity, and 𝜅 = 0.97) [23]. Moreover, our cell-based ELISA system is way better in sensitivity and ICC agreement than the commercialized N-based ELISA (37% sensitivity, 100% specificity, and 𝜅 = 0.37) [23].

Compared with full-length and truncated S protein-based ELISA systems, our cell-based ELISA presents a unique advantage in that the S-displayed cells can directly be attached to the wells of 96-well plates as antigen for ELISA. In contrast, classical indirect ELISA requires the antigen to be purified from samples or engineered cells. The purification process is time-consuming, tedious, and often results in protein loss. When encountering target antigens that are membrane-bound, detergents are required to isolate proteins from membranes, consequently necessitating prolonged dialysis to remove the detergent. The generation of TM-truncated constructs may make purification easier, but protein truncation may disrupt protein conformation and impair antigenicity. By using cell-surface display, we can easily mass produce an ELISA system with full-length S antigen. Simply comparing the amount of cell culture medium required to prepare a 96-well plate (approximately 20 μg per 96-well plate) [23], the full-length S-based ELISA produced from HEK-293T cells by protein purification requires 10 mL, whereas our cell-based ELISA using insect cells requires only 2 mL without the need for protein purification.

Preservation of full-length S with the correct conformation may be particularly crucial for G2b PEDV detection. We found that serum from most of the SPF-I piglets failed to hybridize with S in our Western blot analysis (Figure 3), despite our IFA and cell-based ELISA confirming the presence of anti-S antibodies in those sera (Figure 4D and Figure 7). This outcome is similar to that observed from the neutralizing mAb P4B. P4B recognized S protein displayed on Sf21 cell membranes (Figure 5C), but it could not recognize S in the Western blot analysis (Figure 5B). Since the antigens in the Western blot were wholly or partially denatured, but the antigens in IFA were mainly in their native states, these results indicate that swine tend to generate high levels of conformation-specific anti-S antibodies upon PEDV infection. In previous studies of PEDV mAb [25,26], neutralizing antibodies were primarily found to target conformational epitopes to neutralize the cell entry function of S. Similar to our P4B results, hybridization of these neutralizing antibodies with S was only successful in IFA [25,26]. Unlike mAb, the serum of PEDV-infected swine is composed of polyclonal antibodies that target different epitopes. Our study indicates that the majority of these anti-S antibodies are still conformation-dependent. Therefore, in serological assays, it is important that the antigen is a full-length construct of S in the native conformation as it contains all the conformational epitopes for antibody recognition. Accordingly, our cell-based ELISA may represent a more effective means of evaluating PEDV infections and effective vaccinations (depending on neutralizing antibody) against this highly fatal disease.

Coronaviruses are a massive concern for human health and livestock. Other than PEDV of swine, human coronaviruses (such as SARS-CoV, MERS-CoV, and especially SARS-CoV-2) have also caused severe epidemics or pandemics in humans. ELISA is a convenient and sensitive method for detecting such infections. Our cell-based ELISA system (summarized in Figure 1) represents a straightforward yet more efficient strategy for identifying S-specific antibodies, as exemplified here for PEDV infection in swine. Through automation, the system can potentially be used to test hundreds to thousands of samples in response to the appearance of large numbers of patient serum specimens every day. In addition to coronaviruses, many other viral diseases are emerging in the world. We believe this cell-based ELISA system could be applied to virtually all other viral antigens for better serum detection.

## Figures and Tables

**Table 1:** Statistical analyses of serological results for each group.

Groups	No. of Pigs	ICC Staining		S-Bac Infected Cell-Based ELISA			
				(Cutoff Mean+2SD)		(Cutoff Mean+3SD)	
		+	−	+	−	+	−
SPF-N	14	0	14	0	14	0	14
SPF-I	14	14	0	14	0	14	0
C-N	23	0	23	2	21	1	22
C-I	23	23	0	23	0	23	0
Sow	26	26	0	26	0	26	0
Sensitivity		100%		100%		100%	
Specificity		100%		95%		97%	
𝜅		1		0.96		0.98	

(+): positive; (−): negative; SPF-N: specific pathogen-free PEDV-negative piglets; SPF-I: PEDV-inoculated SPF piglets; C-N: conventional farm-based PEDV-negative piglets; C-I: PEDV-inoculated conventional piglets; Sow: farm-based sows fed with PEDV-positive intestinal homogenate.

## Data Availability

We will make data fully available and without restriction.
